# Loss of Elp3 Impairs the Acetylation and Distribution of Connexin-43 in the Developing Cerebral Cortex

**DOI:** 10.3389/fncel.2017.00122

**Published:** 2017-05-01

**Authors:** Sophie Laguesse, Pierre Close, Laura Van Hees, Alain Chariot, Brigitte Malgrange, Laurent Nguyen

**Affiliations:** ^1^GIGA-Neurosciences, University of LiègeLiège, Belgium; ^2^Interdisciplinary Cluster for Applied Genoproteomics (GIGA-R), University of LiègeLiège, Belgium; ^3^GIGA-Molecular Biology of Diseases, University of LiègeLiège, Belgium; ^4^Walloon Excellence in Lifesciences and Biotechnology (WELBIO)Wallonia, Belgium

**Keywords:** acetylation, cerebral cortex, connexin-43, elongator, HDAC6

## Abstract

The Elongator complex is required for proper development of the cerebral cortex. Interfering with its activity *in vivo* delays the migration of postmitotic projection neurons, at least through a defective α-tubulin acetylation. However, this complex is already expressed by cortical progenitors where it may regulate the early steps of migration by targeting additional proteins. Here we report that connexin-43 (Cx43), which is strongly expressed by cortical progenitors and whose depletion impairs projection neuron migration, requires Elongator expression for its proper acetylation. Indeed, we show that Cx43 acetylation is reduced in the cortex of Elp3cKO embryos, as well as in a neuroblastoma cell line depleted of Elp1 expression, suggesting that Cx43 acetylation requires Elongator in different cellular contexts. Moreover, we show that histones deacetylase 6 (HDAC6) is a deacetylase of Cx43. Finally, we report that acetylation of Cx43 regulates its membrane distribution in apical progenitors of the cerebral cortex.

## Introduction

The neocortex is a highly organized structure made of six distinct neuronal layers, which differ in terms of connectivity and gene expression profile (Molyneaux et al., 2007). The establishment of the laminated cortical structure implies a coordinated generation, migration and differentiation of neurons during embryonic development (Noctor et al., 2001). Excitatory projection neurons arise from progenitor cells located in the ventricular (VZ) and subventricular zones (SVZ) of the dorsal telencephalon (Götz and Huttner, 2005). Newborn neurons migrate radially along radial glial fibers to the cortical plate (CP) and settle into neuronal laminae. Neuronal migration is a dynamic and highly regulated process including distinct phases associated with specific morphologies (Ohtaka-Maruyama and Okado, 2015). Numerous molecular pathways controlling neuronal migration have been identified, including cytoskeletal regulators such as doublecortin, filamin A, and Lis1 (Kriegstein and Noctor, 2004; Liu, 2011; Moon and Wynshaw-Boris, 2013). We previously reported that Elongator is a critical player in the control of cortical neuron migration (Creppe et al., 2009; Tielens et al., 2016). Elongator is a macromolecular complex composed by two copies of six individual subunits (Glatt et al., 2012), with Elp3 being the enzymatic core that contains an acetyltransferase (HAT) domain (Winkler et al., 2002). Several functions have been attributed to Elongator (Nguyen et al., 2010; Glatt and Müller, 2013). Besides its central role as a tRNA-modifying protein (Ladang et al., 2015; Laguesse et al., 2015; Delaunay et al., 2016), Elp3 has been shown to regulate the acetylation of three proteins: histone H3 in the nucleus (Winkler et al., 2002), bruchpilot at the drosophila neuromuscular junction (Miskiewicz et al., 2011) and α-tubulin in migrating post-mitotic projection neurons (Creppe et al., 2009). We previously showed that reducing Elongator activity in post-mitotic neurons correlates with reduced α-tubulin acetylation and impaired migration to the CP (Creppe et al., 2009). However, as Elongator subunits are also found in the VZ/SVZ of the developing cortex (Creppe et al., 2009; Laguesse et al., 2015), we hypothesized that other targets of Elongator (direct or indirect) play a role in the regulation of early steps of migration, contributing to the overall migration impairment observed after depletion of Elongator.

The GAP junction protein connexins (Cx) are highly expressed in neural progenitor cells during cortical development where they regulate different aspects of neurogenesis (Sutor and Hagerty, 2005; Elias and Kriegstein, 2008; Orellana et al., 2013). Among them, connexin-43 (Cx43) is highly expressed by neurons and neuronal progenitors during development (Rouach et al., 2002). Cx43 controls the differentiation and the interkinetic nuclear migration of neuronal progenitors (Liu et al., 2010; Santiago et al., 2010; Rinaldi et al., 2014), the tangential to radial migratory switch of migrating interneurons invading the cortical wall (Elias et al., 2010), and the radial migration of projection neurons (Elias et al., 2007; Elias and Kriegstein, 2008; Cina et al., 2009; Liu et al., 2012; Qi et al., 2016). During radial migration, Cx43 is expressed at the contact point between migrating neurons and radial glial fibers, and targeting Cx43 impairs neuronal migration (Elias et al., 2007; Cina et al., 2009). Cx43 is regulated by numerous post-translational modifications (Solan and Lampe, 2009; Johnstone et al., 2012) including lysine acetylation, which controls its subcellular localization in mouse cardiomyocytes (Colussi et al., 2011).

Here, we showed that Cx43 interacts with both Elp1 and Elp3 in the developing mouse cortex as well as in different cell lines, and that proper acetylation of Cx43 requires Elongator activity, a post-translational modification removed by histones deacetylase 6 (HDAC6). We thus investigated the possible function of Cx43 acetylation and demonstrated that this post-translational modification regulates Cx43 cellular localization in Hela cells and in the developing cortex.

## Materials and Methods

### Animals

Time-pregnant NMRI (Janvier Labs, Saint Berthevin, France), FoxG1^cre/WT^ and Elp3^loxp/loxp^ mice backcrossed in 129/SvJ genetic background were housed under standard conditions. This study was carried out in accordance with the recommendations of the guidelines of the Belgian Ministry of Agriculture in agreement with European Community Council Directive for the care and use of laboratory animals of 22 September 2010 (2010/63/EU) and approved by the local ethics committee. The generation of the conditional Elp3 knock-out mouse required breeding of Elp3^loxp/loxp^ mice with FoxG1^cre/WT^ mice (Hébert and McConnell, 2000), as previously described (Laguesse et al., 2015). The following primers were used for genotyping FoxG1 and Cre recombinase: 5′-GCC GCC CCC CGA CGC CTG GGT GAT-3′, 5′-TGG TGG TGG TGA TGA TGA TGG TGA TGC TGG-3′ and 5′-ATA ATC GCG AAC ATC TTC AGG TTC TGC GGG-3′.

### RNA Extractions and qRT-PCR

Total RNA was extracted from cortices of E14.5 embryos. RNA extraction was performed using the All prep DNA/RNA/protein kit (Qiagen, Hilden, Germany). All RNA samples were treated with DNAse I (Roche, Basel, Switzerland). Synthesis of cDNA was performed on total RNA, which was reverse-transcribed with SuperScript III reverse transcriptase (ThermoFisher Scientific, Waltham, MA, USA) according to the manufacturer’s instructions. Resulting cDNA was used for quantitative PCR, using Faststart Universal SYBR Green Master (Roche). Thermal cycling was performed on an Applied Biosystem 7900HT Fast Real-Time PCR detection system (Applied Biosystems, Foster city, CA, USA). The quantity of each mRNA transcript was measured and expressed relative to Glyceraldehyde-3-Phosphate deshydrogenase (*GAPDH*). The following primers were designed with Primer3 software: *GJA1* forward 5′-GGA CTG CTT TCT CTC ACG TC-3′ and *GJA1* reverse 5′-GAG CGA GAG ACA CCA AGG AC-3′; *GAPDH* forward 5′-GCA CAG TCA AGG CCG AGA AT-3′ and *GAPDH* reverse 5′-GCC TTC TCC ATG GTG GTG AA-3′.

### Cell Cultures, Stable Line Establishment and Transfections

Human Glioblastoma (U87) and Adenocarcinoma (Hela) cells were cultured in DMEM medium supplemented with Bovine fetal serum (FBS) 10%. Mouse Neuroblastoma cells (N2A) were cultured in DMEM supplemented with FBS 10% and glutamine 2 mM. Human embryonic kidney HEK-293 lentiX cells (Clontech, Moutain View, CA, USA) and HEK-293 cells were cultivated in DMEM supplemented with FBS 10%, glutamine 1%. To generate HEK-293 stably expressing ELP3-FLAG proteins, cells were transfected with pIRES-Elp3-puro construct and selected in 1 μg/ml puromycin (Sigma Aldrich, St Louis, MO, USA). Cells were maintained in selecting media for 3 weeks and surviving cells were used for experiment after transgene expression confirmation. Cell transfections were performed using lipofectamine 2000 according to manufacturer’s protocol (ThermoFisher Scientific). Cells were lysed or fixed 48 h after transfection. Trichostatin A (TSA; 5 mM in DMSO, Sigma-Aldrich) was added to the medium at a final concentration of 1 μM for 4 h before cell fixation or lysis.

### Plasmids Constructs and Preparation

ORFs encoding human Elp3 were cloned into pIRESpuro (Clontech) with a FLAG tag at the C terminus. Flag-HDAC6 in pcDNA3 has been described previously (Viatour et al., 2003). All constructs were sequence verified. Cx43 was subcloned from the clone image MC205621 (Origene, Rockville, MD, USA) by high fidelity PCR using NheI-Cx43 forward primer and EcoRV-reverse primer and inserted into the pCAGGS-IRES-GFP vector. To obtain Cx43-4KR, directed mutagenesis was performed on the pCAGGS-Cx43-IRES-GFP (Agilent, Santa Clara, CA, USA), replacing K9R, K162R, K234R and K264R. Plasmids DNA were prepared using a Plasmid Endofree Maxi Kit (Qiagen).

### Lentivirus Production and Infection

Lentivirus production and lentiviral infections were performed as previously described (Creppe et al., 2009). Briefly, HEK-293 lentiX cells were transfected with the lentiviral packaging vectors VSVG and R8.91 and the pLL3.7 shELP1 or pLL3.7 shSCR using Fugene6 (Promega, Madison, WI, USA) in Opti-MEM medium. Twenty-four hours after transfection, medium was changed to DMEM-FBS 10%. Seventy-two hours after transfection, supernatant containing the viral particles was collected and passed through 0.22 μm filter. The supernatant was then used to infect N2A cells two times consecutively for 6 h with Polybrene® (Sigma-Aldrich) added at 5 μg/mL. Efficacy of infection was determined by GFP expression.

### Immunohistochemistry

Embryonic brains (E14.5) were dissected in 0.1 M phosphate-buffered saline pH7.4 (PBS) and were fixed at 4°C in 4% paraformaldehyde (PFA) for 1 h. Fixed samples were cryoprotected overnight in 20% sucrose in PBS at 4°C, embedded in OCT Compound (VWR International, Leuven, Belgium) and sectioned (12 μm) onto slides (SuperFrost Plus, VWR International) using a cryostat. Cells were fixed at RT in 4% PFA for 15 min and rinsed three times with PBS. Frozen cryosections and fixed cells were washed three times in PBS-Triton 0.1% (PBST) and blocked for 1 h at room temperature in PBST containing 10% donkey serum (Jackson Immunoresearch Laboratories, West Grove, PA, USA). Sections were incubated overnight at 4°C with the following primary antibodies: anti-Elp3 (1:1000, gift from J. Svejstrup, Cancer Research UK London Research Institute, South Mimms, UK), anti-Cx43 (1:500, rabbit, Abcam, Cambridge, UK), anti-Cx43 IF1 (1:500, mouse, Max Planck institute, Munchen, Germany, Sosinsky et al., 2007), anti-GFP (1:500, goat, Abcam). After washing, sections were incubated for 1 h at room temperature with either anti-mouse, anti-rabbit, or anti-goat secondary antibodies coupled to Rhodamine-redX or FITC (Jackson Immunoresearch Laboratories). Nuclei were counterstained with Hoechst 33342 (1:1000, ThermoFisher Scientific), washed in PBST and coverslipped using Aqua Polymount (Polysciences Inc, Washington, DC, USA). The slides were stored in the dark at 4°C. For images analyses, sections were analyzed by confocal microscopy using A1Ti confocal microscope (Nikon) and ImageJ software. Images of cortical slices were acquired with a 40× objective with a *z*-interval of 1 μm (*z*-stack = 5 images) or with a 60× objective with a *z-interval* of 1 μm (*z*-stack = 5 images). The quantifications of intracellular and membrane fluorescent signal intensities in Hela cells were conducted using ImageJ software.

### Immunoprecipitation and Western Blot Analysis

Dissected E14.5 embryonic cortices were incubated in lysis buffer (50 mM Tris-HCl pH 7.4, 450 mM NaCl, 1% triton, 10 mM NaF, 1 mM Na_3_VO_4_ and proteases inhibitors, Roche). Cells were lysed in another lysis buffer (50 mM Tris-HCl pH 7.4, 150 mM NaCl, 1% triton, 10 mM NaF, 1 mM Na_3_VO_4_ and proteases inhibitors, Roche). Proteins were extracted by centrifugation (10,000 *g*) for 10 min at 4°C, and quantified using BCA method (Pierce). Immunoprecipitation (IP) was carried out using the following antibodies at 1/250: anti-HA (rabbit, Santa Cruz Technology SCT, Santa Cruz, CA, USA), anti-HA (mouse, SCT), anti-Flag (mouse, Sigma-Aldrich), anti-ELP1 (Close et al., 2006), anti-ELP3 (gift from J. Svejstrup, Cancer Research UK London Research Institute, South Mimms, UK), anti-Cx43 (rabbit, Abcam), followed by 1 h incubation with protein A/G plus agarose beads. Anti-HA IP was carried out as control. Beads were washed out six times with the lysis buffer. Protein samples were then mixed with loading buffer and incubated at 95°C for 5 min, then separated by SDS-PAGE and transferred to 0.45 μm PVDF membranes (Millipore, Billerica, MA, USA). Membranes were blocked 1 h in a solution containing non-fat milk, then incubated O/N at 4°C with the following antibodies anti-ELP1 (Close et al., 2006), anti-Cx43 (Abcam), anti-pan-acetylated lysine (Cell signaling technology, Danvers, MA, USA). HRP-conjugated antibodies were applied for 1 h at RT (conjugated anti-mouse, anti-rabbit, GE Healthcare, Waukesha, WI, USA). Membranes were developed with the ECL chemiluminescent reagent (Thermo scientific, Rockford, IL, USA) using Hyperfilm ECL (GE Healthcare). ImageJ was used for optical density quantification.

### Statistical Analyses

Statistics for dual comparisons were generated using unpaired two-tailed Student’s *t*-tests. Statistical analyses were performed using graphPad Prism 5.0 Software (GraphPad software Inc., San Diego, CA, USA). Values are presented as mean ± SEM *p < 0.05, **p < 0.01, ***p < 0.001 for all statistics.

## Results

### Elongator Interacts with Connexin 43 in the Developing Cortex

**Figure 1:**
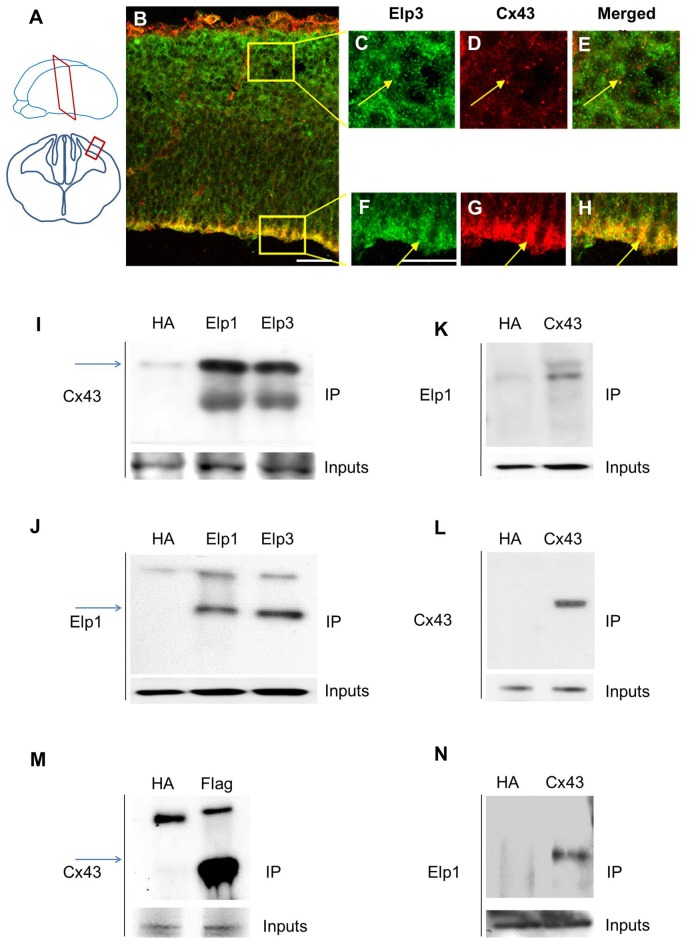
**Elongator interacts with connexin 43 (Cx43) in the developing cortex. (A)** Scheme illustrating the location of the coronal section (12 μm) of E14.5 wild-type (WT) cortex. **(B–H)** Immunodetection of Cx43 (red) and Elp3 (green) in E14.5 WT cortex showing co-expression of both Elp3 and Cx43 in cortical plate (CP) neurons **(C–E)** and in the ventricular (VZ)/subventricular zones (SVZ) neuronal progenitors **(F–H)**. **(I–L)** Immunoprecipitates from E14.5 mouse embryos cortices were subjected to anti-ELP1 or anti-Cx43 western blot analysis; corresponding western blots were performed on crude cell extracts (inputs). **(M)** Immunoprecipitates from HEK293 cell line stably expressing flag-ELP3 were subjected to anti-Cx43 western blot analysis and showed an interaction between Cx43 and ELP3. Corresponding western blots were performed on crude cell extracts (inputs). **(N)** Forty-eight hours after transfection of Cx43 in N2A cells, immunoprecipitates from N2A cells homogenate were subjected to anti-ELP1 western blot and showed an interaction between Cx43 and ELP1. Bar scale, 50 μm.

Cx43 is expressed in neurons and progenitors of the developing cortex of the rat brain, with high levels of expression in the VZ/SVZ and reduced levels in the CP (Elias et al., 2007; Qi et al., 2016). We analyzed the expression of Cx43 in E14.5 embryonic mouse brain and observed a punctate staining throughout the cortical wall, with a stronger labeling of the VZ cells (Figures 1A–H). The different subunits of Elongator are expressed in migrating projection neurons, but are also found in the VZ/SVZ progenitor cells (Creppe et al., 2009; Laguesse et al., 2015). We thus immunolabeled embryonic cortices to detect Cx43 and Elp3, and we observed a strong co-expression of both proteins in cortical progenitor cells that are lining the ventricle (Figures 1B,F–H). Co-IP experiments on microdissected tissue from the cerebral cortex of E14.5 embryos demonstrated an *in vivo* interaction between Cx43 and both Elp1 and Elp3 (Figures 1I–L). We also generated a HEK293 cell line stably expressing flag-Elp3, and we detected a comparable interaction between flag-ELP3 and Cx43 *in vitro* (Figure 1M). This interaction was further confirmed in a neuroblastoma cell line (Figure 1N).

### Connexin 43 is Acetylated in the Developing Cortex

**Figure 2:**
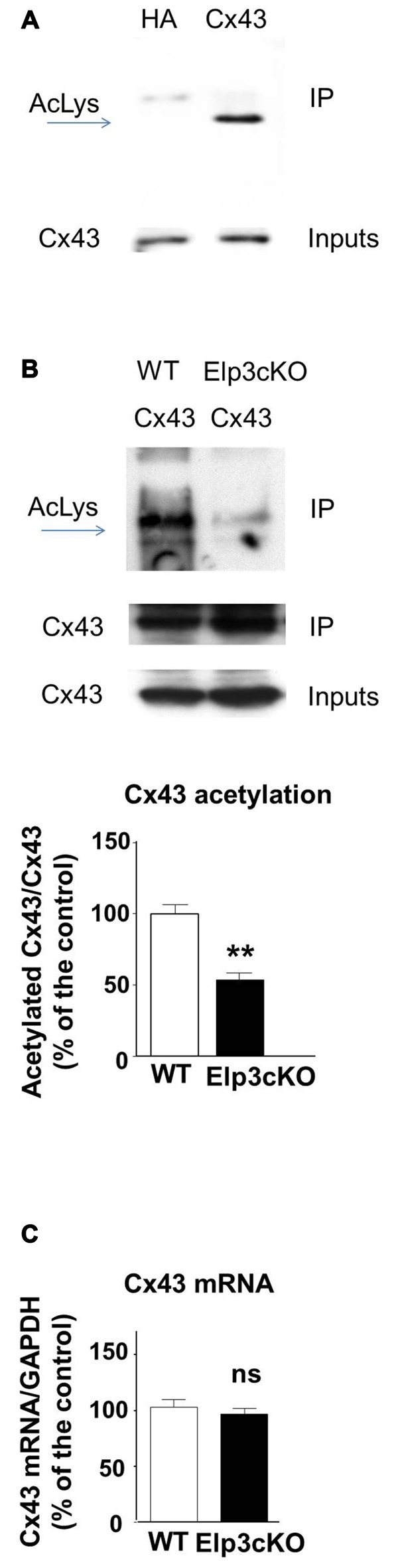
**Cx43 is acetylated in the developing cortex. (A,B)** Immunoprecipitates from E14.5 WT **(A)** or Elp3cKO **(B)** mouse embryos cortices were subjected to anti- pan acetyl lysine western blot analysis. Corresponding western blots were performed on crude cell extracts (inputs). Data are presented as the average ratio of acetylated Cx43 to Cx43 ± SEM, and are expressed as percentage of WT controls. Significance was determined using two-tailed unpaired *t*-test *t*_(4)_ = 5.11, *p* = 0.007. *n* = 3. **(C)** *Cx43* mRNA levels were determined by qRT-PCR in E14.5 WT and Elp3cKO cortex. Data are presented as the average ratio of *Cx43* to glyceraldehyde-3-Phosphate deshydrogenase (*GAPDH*) ± SEM, and expressed as percentage of WT control. Significance was determined using two-tailed unpaired *t*-test *t*_(4)_ = 0.668, *p* = 0.54. *n* = 4. **p < 0.01.

In the developing mouse heart, Cx43 assembles into GAP junctions expressed at the intercalated discs that physically delimitate cardiomyocytes, and Cx43 acetylation has been shown to control such localization (Colussi et al., 2011). To determine whether Cx43 is acetylated in the developing mouse cortex, we performed western blot to detect acetylated lysine(s) in Cx43 immunoprecipitate from E14.5 mouse cortical extracts (Figure 2A). The acetylation level of Cx43 but not its expression (Figure 2B (inputs), Figure 2C) was reduced in the cortex of E14.5 Elp3cKO embryos (breeding of Elp3loxlox mice with FoxG1: Cre mice (Hébert and McConnell, 2000), as previously described (Laguesse et al., 2015)), as compared to WT embryos (Figure 2B). Altogether, these results show that Elongator is required for the proper acetylation of Cx43 in the mouse developing cerebral cortex.

### Elongator and HDAC6 Regulate Connexin 43 Acetylation

**Figure 3:**
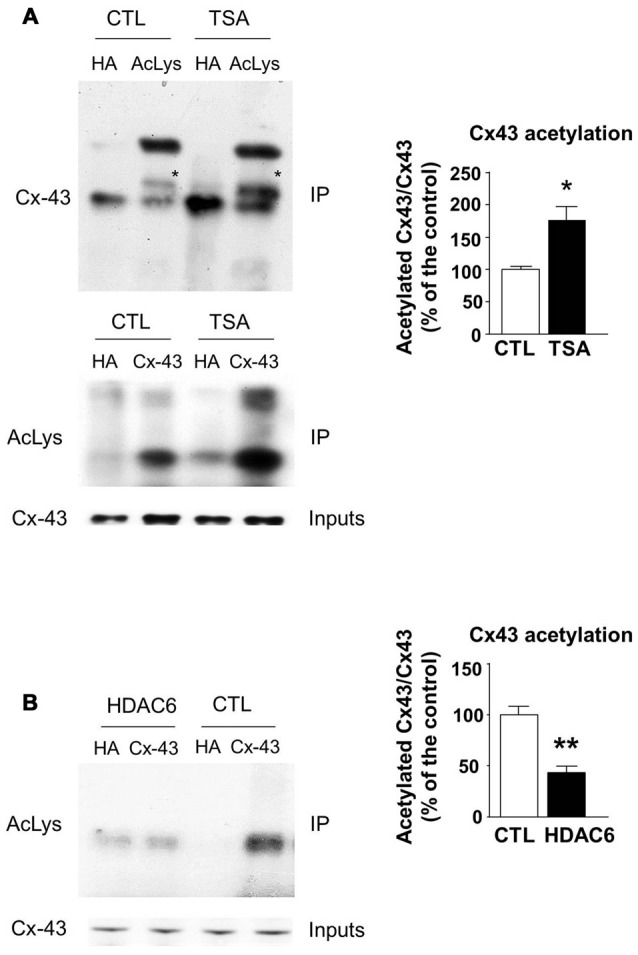
**Histones deacetylase 6 (HDAC6) regulates Cx43 acetylation levels. (A)** U87 cells were treated with trichostatine A (TSA) or DMSO as control (CTL) for 4 h. Immunoprecipitation (IP) were carried out using anti-pan acetyl lysine antibodies or anti-HA as control, followed by western blot analysis using specific anti-Cx43 antibody (upper panel); or Cx43 IP was carried out and followed by western blot using anti-pan acetyl lysine antibody (lower panel). Corresponding western blot were performed on crude cell extracts (inputs). Data are presented as the average ratio of acetylated Cx43 to Cx43 ± SEM, and are expressed as percentage of control. Significance was determined using two-tailed unpaired *t*-test *t*_(4)_ = 3.29, *p* = 0.03. *n* = 3. Stars indicate the band corresponding to Cx43. **(B)** N2A cells were transfected with Cx43 and HDAC6 or the empty plasmid as control (CTL) and were lysed 48 h later. IPs were carried out using anti-Cx43 or anti-HA antibodies and followed by western blot analysis using a pan acetyl lysine antibody. Data are presented as the average ratio of acetylated Cx43 to Cx43 ± SEM, and are expressed as percentage of control. Significance was determined using two-tailed unpaired *t*-test *t*_(4)_ = 5.12, *p* = 0.007. *n* = 3. *p < 0.05; **p < 0.01.

**Figure 4:**
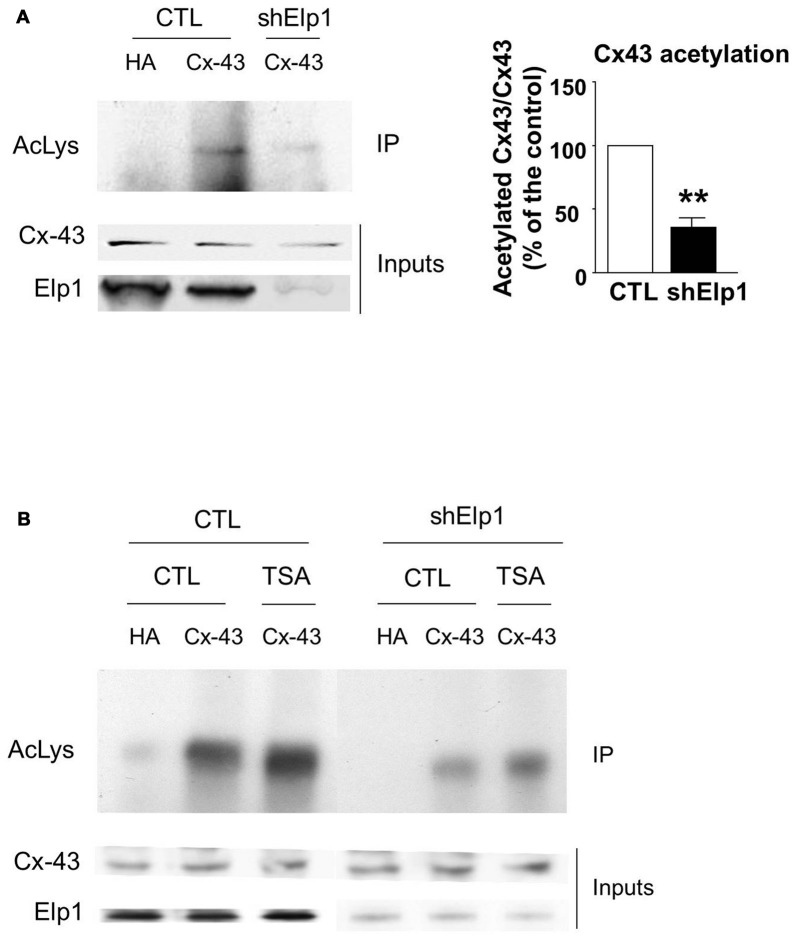
**Elongator depletion results in reduced Cx43 acetylation levels. (A,B)** N2A cells were transfected with Cx43 and were infected 24 h later with Ltv-shElp1 or Ltv-SCR as control (CTL). Seventy-two hours after lentiviral infection, cells were treated with TSA or DMSO for 4 h **(B)** and IP of Cx43 was carried out, followed by western blot using an anti-pan acetyl lysine antibody. Corresponding western blots were performed on crude cell extracts (inputs). Data are presented as the average ratio of acetylated Cx43 to Cx43 ± SEM, and are expressed as percentage of control. Significance was determined using two-tailed unpaired *t*-test *t*_**(3)**_ = 6.77, *p* = 0.007. *n* = 3. **p < 0.01.

The transfer or removal of acetyl groups on lysine residues is mediated by two classes of enzymes: the lysine acetyltransferases (KATs) and lysine deacetylases (KDACs), also known as HDACs (Menzies et al., 2016; Simon et al., 2016). HDACs are grouped into four classes, depending on sequence homology. Classes I, II and IV are Zinc-dependent HDACs, whereas class III consists of NAD^+^-dependent sirtuins (SIRT1 and 2). HDACs play important roles in the regulation of transcription, but they also act on a large set of non-histones proteins, like transcription factors, translation-associated proteins, proteins involved in cytoskeleton regulation or cell signaling, to regulate several functions (Yao and Yang, 2011; Roche and Bertrand, 2016). In order to identify the enzyme responsible for Cx43 deacetylation, we treated cultured U87 cells with TSA, a potent inhibitor of class I, II and IV HDACs but not class III sirtuins (Codd et al., 2009), or DMSO as control. As shown in Figure 3A, TSA treatment of U87 cells significantly increased Cx43 acetylation levels, suggesting that the Cx43 deacetylase is a member of HDAC family. HDAC6 is found in the cytoplasm and promotes the deacetylation of multiple targets including α-tubulin, cortactin and HSP90 (Kovacs et al., 2005; Zhang et al., 2007; Li et al., 2011). We thus tested the ability of HDAC6 to promote Cx43 deacetylation. As shown in Figure 3B, HDAC6 overexpression reduced the acetylation of Cx43 in N2A cells, suggesting that HDAC6 is a deacetylase that targets Cx43. We next deciphered whether Elongator also promotes Cx43 acetylation in N2A cell line. For this purpose, we infected N2A cells with a lentivirus delivering shRNA against ELP1, because it has previously been reported to destabilize the Elongator complex (Petrakis et al., 2004; Close et al., 2006; Creppe et al., 2009). As shown in Figure 4A, ELP1 depletion in N2A cells resulted in decreased Cx43 acetylation level. We further showed that inhibition of HDACs activity was not sufficient to counteract the impact of the depletion of ELP1 on Cx43 acetylation (Figure 4B). Altogether, these results suggest that Elongator is necessary for proper acetylation of Cx43, a post-translational modification removed at least in part by HDAC6.

### Acetylation of Cx43 Regulates its Membrane Localization *In Vitro*

**Figure 5:**
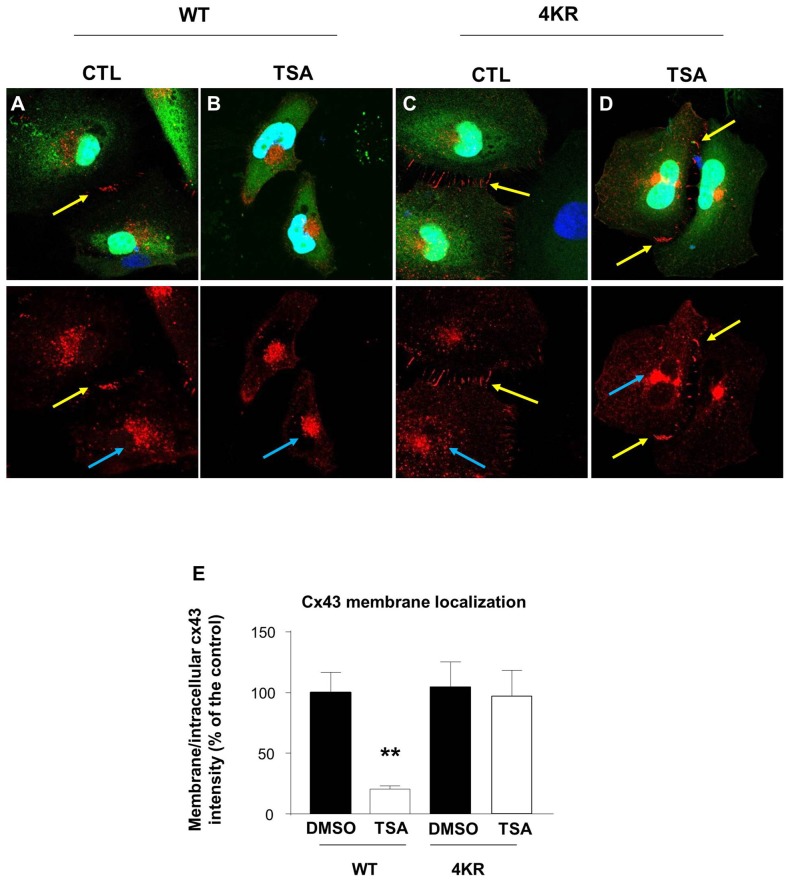
**Acetylation regulates Cx43 localization. (A–D)** Hela cells were transfected with Cx43-WT-GFP **(A,B)** or Cx43-4KR-GFP **(C,D)** and were treated with TSA or DMSO as control (CTL) for 4 h. Immunodetection of Cx43 (red, cx43 rabbit, Abcam) in transfected Hela cells (green, GFP) and DAPI (blue) showed the different cellular localization of Cx43-WT and Cx43-4KR upon TSA treatment. Yellow arrows indicate connexons between two adjacent cells; blue arrows indicate intracellular Cx43 labeling. **(E)** Cx43 membrane localization was determined by measuring the ratio of membrane and intracellular immunofluorescence intensity. **p < 0.01.

It has been previously shown that acetylated Cx43 delocalizes from the membrane and is mainly found in the cytoplasm and in the nucleus (Colussi et al., 2011). We thus analyzed the cellular localization of Cx43 in Hela cells before and after TSA treatment. We overexpressed Cx43 and showed that in basal conditions, Cx43 formed connexons at the contact points between cells (Figure 5A). However, TSA treatment delocalized Cx43 from the membrane to a more cytoplasmic position (Figures 5B,E), in line with previous results (Colussi et al., 2011). As Cx43 contains many lysine residues, we used the posttranslational modification database PHOSIDA to identify potentially acetylable lysines (Gnad et al., 2011). We identified four lysines (K) predicted to be acetylated with more than 90% confidence: K9, K162, K234 and K264. In order to generate a non-acetylable form of Cx43, we replaced the four corresponding lysines by arginine residues (R), as previously described (Li et al., 2002; Qiang et al., 2010; Colussi et al., 2011; Jiménez-Canino et al., 2016). Cx43-4KR was also found in connexons connecting Hela cells (Figures 5C,E), but unlike Cx43-WT, TSA treatment did not trigger its delocalization from the membrane, and Cx43-4KR was still found at the contact points between cells after TSA treatment (Figures 5D,E). This suggests that the cellular localization of Cx43 is regulated through acetylation, which triggers Cx43 delocalization from the membrane.

### Increased Cx43 Membrane Localization in the VZ Progenitor Cells of Elp3cKO

**Figure 6:**
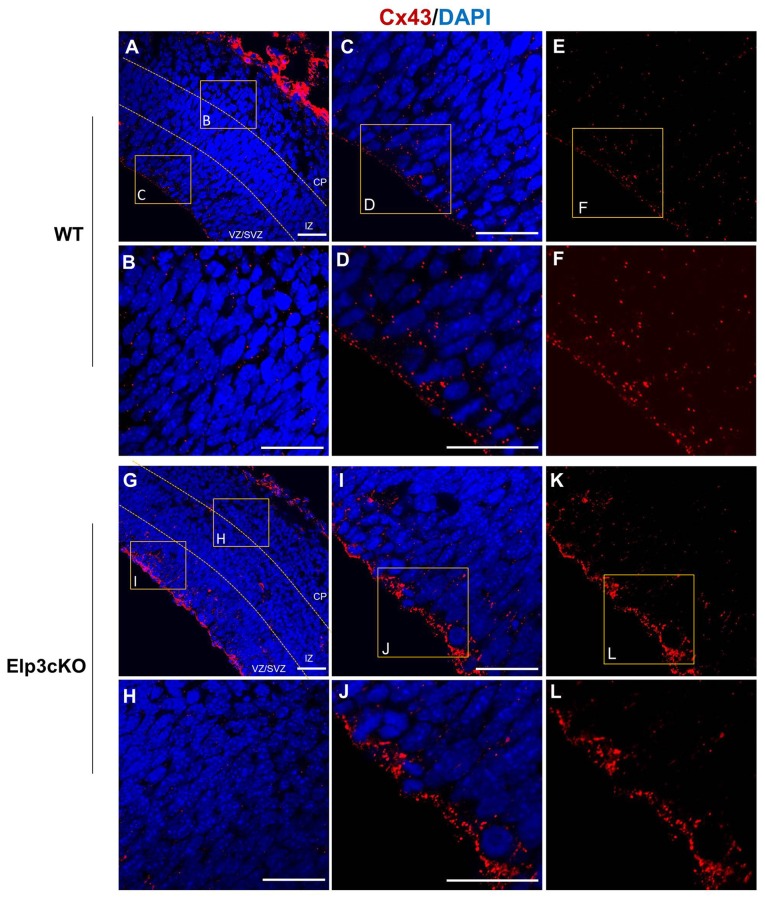
**Cx43 membrane localization is enhanced in Elp3cKO cortical progenitors. (A–L)** Immunodetection of membrane Cx43 (red, Cx43 IF1 antibody) and DAPI (blue) in the cortical wall of WT **(A–F)** and Elp3cKO **(G–L)** E14.5 embryos show increased membrane distribution of Cx43 in the VZ progenitor cells of Elp3cKO embryos compared to WT embryos. Bar scale, 50 μm.

As Cx43 acetylation regulates its subcellular localization and is reduced in Elp3cKO embryonic cortices, we assessed the impact of the loss of Elp3 on Cx43 localization. We labeled Cx43 in E14.5 WT and Elp3cKO cortex with a specific antibody, which detects Cx43 only when present at the membrane (IF1 antibody; Sosinsky et al., 2007) and analyzed its distribution in the cortical wall. In WT cortex, we observed membrane Cx43 labeling throughout the cortical wall with some accumulation detected in the VZ/SVZ, as compared to the IZ and CP (Figures 6A–F). In the Elp3cKO cortex, we observed a reduced Cx43 labeling in the IZ/CP compared to the WT littermate (Figures 6G,H), which is likely the consequence of the reduced neuron population observed upon loss of Elp3 in cortical progenitors (Laguesse et al., 2015). However, as shown in Figures 6I–L, our results showed a stronger labeling of Cx43 in the VZ progenitor cells of Elp3cKO embryos compared to the WT cortex, without change of total Cx43 protein and mRNA expression (Figures 2B,C). This suggests that the reduced acetylation of Cx43 occurring upon loss of Elongator activity promotes its membrane localization.

## Discussion

Here we present evidences showing that Cx43 is acetylated and interacts with Elongator in the developing cortex as well as in several cell lines. We further show that depletion of Elongator *in vivo* impairs the acetylation of Cx43, suggesting that Elp3 promotes Cx43 acetylation in the developing cerebral cortex. We also identified HDAC6 as a deacetylase of Cx43. Finally, our data suggest that the acetylation of Cx43 regulates its localization in cortical progenitors.

We previously reported that Elongator controls the radial migration of projection neurons partly through α-tubulin acetylation (Creppe et al., 2009). However, Elongator is also functionally expressed in cortical progenitor cells, where loss of its activity results in impaired tRNA modification and protein translation, triggering the unfolded protein response that ultimately leads to defects in neurogenesis (Laguesse et al., 2015). Given that Elongator functions in neuronal migration and is expressed in cortical progenitors, we reasoned that Elongator could triggers the early steps of neuronal migration in the projection neuron progenitors. Cx43 is mainly expressed in the VZ/SVZ of the developing cortex and its loss has been reported to delay radial migration of projection neurons (Elias and Kriegstein, 2008; Cina et al., 2009), a phenotype similar to the one observed upon knockdown of Elongator subunits (Creppe et al., 2009). We found that Cx43 interacts with both Elp1 and Elp3 in the developing cortex, as well as in several cell lines. We also showed the existence of a co-expression of Cx43 and Elongator throughout the cortical wall, which is stronger in neuronal progenitors lining the lateral ventricle. This suggests a potential regulation of Cx43 by Elongator in the VZ/SVZ of the developing cortex.

Cx43 is regulated by several post-translational modifications, including phosphorylation (Solan and Lampe, 2009; Alstrom et al., 2015), ubiquitination (Ribeiro-Rodrigues et al., 2015; Leithe, 2016), nitrosylation (Johnstone et al., 2012; Lohman et al., 2016) and acetylation (Colussi et al., 2011; Meraviglia et al., 2015). These modifications regulate a great variety of biological processes, including degradation, changes in binding partners or subcellular localization. We showed that Cx43 is acetylated in the developing cortex, and that its acetylation is reduced in the cortex of Elp3cKO embryos. Our results suggest a new role for Elp3 in the regulation of Cx43 acetylation *in vivo*, which is further supported by results obtained in cell lines. Thus, along with α-tubulin in migrating projection neurons (Creppe et al., 2009) and bruchpilot at the pre-synaptic density of the *Drosophila* neuromuscular junctions whose acetylation is necessary for proper structure of the synapse (Miskiewicz et al., 2011), we suggest that Cx43 is another putative target of Elp3 in VZ/SVZ progenitors of the developing cortex. However, whether Cx43 acetylation is directly regulated by the acetyltransferase activity of Elp3, or indirectly via the upregulated unfolded protein response observed in cortical progenitors of Elp3cKO embryos (Laguesse et al., 2015) is still an open question.

Interestingly, Cx43 acetylation in cardiomyocytes requires the activity of the histone acetyltransferase p300/CBP-associated factor (PCAF), which is a member of the Gcn5-related N-acetyltransferase (GNAT) HAT family that also comprises Elp3 (Sterner and Berger, 2000). Similar to Elp3, PCAF has been shown to acetylate non-histones proteins such as β-catenin (Ge et al., 2009), Akt1 (Zhang et al., 2015), Stat3 (Cai et al., 2014) and lin28 (Wang et al., 2014). It would be interesting to test whether PCAF is also involved in the regulation of Cx43 acetylation in the developing cortex. On the other hand, we showed that Cx43 acetylation is increased upon TSA treatment, suggesting that Cx43 deacetylation depends on HDACs, and we further identified HDAC6 as one deacetylase of Cx43. HDAC6 has a cytoplasmic localization and has been shown to acetylate α-tubulin, as well as other cytoplasmic targets such as HSP90, Cortactin or β-catenin (Li et al., 2011; Yao and Yang, 2011). Interestingly, Colussi et al. (2011) showed a constitutive association of HDAC3, -4 and -5 with Cx43 in cardiomyocytes, and a co-localization at the membrane but also in cytoplasmic and nuclear compartments. These HDACs are known to dynamically shuttle between the nucleus and the cytoplasm in a signal-dependent manner (Li and Yang, 2016). It would thus be of interest to test whether these HDACs could also regulate Cx43 acetylation in the developing cortex.

Phosphorylation and acetylation of Cx43 have been shown to regulate its cellular localization (Sosinsky et al., 2007; Colussi et al., 2011; Qi et al., 2016). Indeed, in migrating neurons, Cx43 phosphorylation at Ser^279^ and Ser^282^ has been found to block its membrane expression and to promote proteasome-dependent degradation (Qi et al., 2016). In cardiomyocytes, Cx43 acetylation leads to its delocalization from the membrane toward intracellular compartment (Colussi et al., 2011). In line with the latter result, we showed in Hela cells that Cx43-WT was present at the membrane under normal conditions, but delocalized from the membrane following TSA treatment. The non-acetylable Cx43-4KR was also found at the membrane in normal conditions, but TSA treatment did not modify its cellular localization, strengthening the idea that Cx43 acetylation regulates its subcellular localization. Our data also showed an excessive membrane localization of Cx43 in the VZ progenitor cells upon loss of Elp3, suggesting that Elp3-dependent acetylation regulates Cx43 cellular localization in neuronal progenitor cells during cortex development.

Cx43 is a GAP junction subunit expressed in many cell types and tissues (Oyamada et al., 2005). Six Cx43 monomers associate to form hexameric hemichannels, also called connexons, which can combine with connexons on adjacent cells to form GAP junctions allowing the exchange of small molecules and ions (Laird, 2006). Besides the communication properties of GAP junctions, it has been reported that connexons can transport ATP and assist in calcium signaling (Goodenough and Paul, 2003) and also promote adhesion in migrating projection neurons (Lin et al., 2002; Elias et al., 2007, 2010; Cotrina et al., 2008; Elias and Kriegstein, 2008). Specifically, it has been shown that Cx43 is necessary for radial migration of projection neurons as well as for the tangential to radial migratory switch in migrating interneurons, and that this function of Cx43 is mediated by adhesion properties of GAP junctions/connexon rather than their channel function (Elias et al., 2007, 2010; Kameritsch et al., 2012). It would thus be interesting to test whether Cx43 acetylation controls the adhesion properties of connexons between cortical progenitor cells or between migrating neurons and radial glia, and determine if replacing the endogenous Cx43 with Cx43-4KR would lead to adhesion and/or migration defects.

## Author Contributions

SL and LN designed the experiment. SL performed the experiment and SL and PC analyzed the data. PC generated the flag-Elp3 cell line. SL and LN wrote the manuscript. BM, AC and PC edited the manuscript. LVH participated in the revisions. All authors discussed the results and implications and commented on the manuscript.

## Conflict of Interest Statement

The authors declare that the research was conducted in the absence of any commercial or financial relationships that could be construed as a potential conflict of interest.
